# Population preferences for breast cancer screening policies: Discrete choice experiment in Belarus

**DOI:** 10.1371/journal.pone.0224667

**Published:** 2019-11-01

**Authors:** Olena Mandrik, Alesya Yaumenenka, Rolando Herrero, Marcel F. Jonker

**Affiliations:** 1 Section of Early Detection and Prevention, International Agency for Research on Cancer, Lyon, France; 2 Erasmus School of Health Policy & Management, Erasmus University Rotterdam, Rotterdam, The Netherlands; 3 The University of Sheffield, School of Health and Related Research (ScHARR), Health Economic and Decision Science (HEDS), Sheffield, the United Kingdom; 4 N.N. Alexandrov National Cancer Center of Belarus, Cancer control department, N.N. Alexandrov National Cancer Centre of Belarus, Liasny, Belarus; 5 Duke Clinical Research Institute, Duke University, Durham, United States of America; 6 Erasmus Choice Modelling Centre, Erasmus University Rotterdam, Rotterdam, The Netherlands; Universidad de Murcia, SPAIN

## Abstract

**Background:**

Reaching an acceptable participation rate in screening programs is challenging. With the objective of supporting the Belarus government to implement mammography screening as a single intervention, we analyse the main determinants of breast cancer screening participation.

**Methods:**

We developed a discrete choice experiment using a mixed research approach, comprising a literature review, in-depth interviews with key informants (n = 23), “think aloud” pilots (n = 10) and quantitative measurement of stated preferences for a representative sample of Belarus women (n = 428, 89% response rate). The choice data were analysed using a latent class logit model with four classes selected based on statistical (consistent Akaike information criterion) and interpretational considerations.

**Results:**

Women in the sample were representative of all six geographic regions, mainly urban (81%), and high-education (31%) characteristics. Preferences of women in all four classes were primarily influenced by the perceived reliability of the test (sensitivity and screening method) and costs. Travel and waiting time were important components in the decision for 34% of women. Most women in Belarus preferred mammography screening to the existing clinical breast examination (90%). However, if the national screening program is restricted in capacity, this proportion of women will drop to 55%. Women in all four classes preferred combined screening (mammography with clinical breast examination) to single mammography. While this preference was stronger if lower test sensitivity was assumed, 28% of women consistently gave more importance to combined screening than to test sensitivity.

**Conclusion:**

Women in Belarus were favourable to mammography screening. Population should be informed that there are no benefits of combined screening compared to single mammography. The results of this study are directly relevant to policy makers and help them targeting the screening population.

## Introduction

Breast cancer is the most common cancer in women worldwide, and breast cancer screening (BCS) programs via mammography are frequently established in an effort to decrease mortality. Reaching high screening uptake is challenging to achieve. Only half of the BCS programs in European Union reached the benchmark of acceptable participation (>70%) [1]. Besides, informed by the ongoing debates on the benefits/harms ratio of BCS [2], women invited to the new screening programs may be negatively affected by the risk of false-positive result of the test and overdiagnosis [3]. This fear of screening-related harms may avert their preferences from BCS in general or shift them towards programs with lower risk of harms from a population perspective (e.g. more qualified doctors, specialized or private hospitals, etc.).Therefore, implementation of any BCS requires a deep insight into population preferences for screening strategies and their characteristics before nation-wide program implementation.

There is limited knowledge about women’s preferences to BCS; these preferences are impacted by cultural and social barriers, including cultural norms, socio-economic status and gender equality index. Thus, the screening preferences will vary among culturally—diverse jurisdictions [4, 5]. Meanwhile, the literature on preferences to BCS among women from low- and middle-income settings is scarce, particularly the documentary sources pertaining to Central and Eastern Europe [6].

The Republic of Belarus (henceforth Belarus) is a middle-income country in Eastern Europe with population of 9.5 million people and total gross domestic product of 188 billion USD (2018) [7]. Since its independence in 1991, Belarus maintained an opportunistic clinical breast examination (CBE) program as part of an annual physical examination. In 2014 the government of Belarus launched several pilot projects aimed at introducing nationwide mammography screening for 50–69 year old women [8]. The mammography screening is provided to the population free of charge similar to the other health services, and the BCS program escalation is planned after proper evaluation of the pilots [9].

Considering the desire of the government in Belarus to implement a nationwide mammography screening, we aimed to support this intention by defining the design of the BCS programs that would get more support from the target population. To do this, we set an objective to assess women’s preferences for the BCS programs using discrete choice experiment (DCE) on a representative sample of women in Belarus. We also aim to demonstrate how providers of BCS services can use empirical findings to develop preferred participation strategies.

## Methods

### Planning the discrete choice experiment

Multiple approaches towards evaluation of respondents’ preferences have been developed through the years. One of them, DCE, is a well-established and Nobel-prize winning preference elicitation technique with a theoretical and behavioural foundation in Random Utility Theory (RUT). RUT assumes that goods and services can be described by their characteristics (called “attributes” in a DCE) and different values of these characteristics (called “levels” in a DCE). By applying the DCE one considers that any health intervention can be described by a combination of levels and that consumer’s utility for these interventions is a function of a combination of these characteristics [10–12]. The strength of DCEs is that they focus on utilities of characteristics of an intervention rather than the general utility of intervention. By doing this, it allows researchers to evaluate preferences of a population to a hypothetical screening program prior to its implementation, and so supports the development of a better and more successful screening design prior to the actual implementation.

Designing a DCE involves a process of developing, testing and optimizing the experiment questionnaire. To develop a DCE a mixed research approach was used, comprising a literature review, exploratory in-depth interviews with the key informants (n = 23), “think aloud” (TAL) pilots of the instrument (n = 10) and quantitative measurement of stated preferences. The protocol and relevant study materials were developed in English, then translated into Russian and verified by the second co-author. Ethics approval of the research protocol was obtained from the International Agency for Research on Cancer (№17–11) and N.N. Alexandrov National Cancer Center of Belarus (№138).

### Selection of attributes and levels

The selection of attributes and levels was based on a step-wise bottom-up approach. As a first step, through an extensive literature review of PubMed, Embase, Scopus and the regional databases (IMSEAR, Index Medicus for the Eastern Mediterranean Region, LILAC/IBECS) in English, Russian, French, Portuguese and Spanish languages we identified 383 abstracts which led to inclusion of 34 full-texts (refer to the S1 File for the detailed protocol including the search strategy and the results). Content analysis of this literature using Atlas.ti software identified 21 plausible factors impacting the decision of women to attend BCS in low- and middle-income countries (Table 1).

**Table 1:** Factors affecting preferences for breast cancer screening: The results of the literature review.

Procedure	Organization	Population and provider
Approach: breast cancer detection strategy and its frequency	Facility: type of facility where the test was preformed	Personal attitude and beliefs of women
Discomfort: discomfort or pain during the test, screening time	Affordability: screening costs, access to free treatment in case the disease is identified)	Socio-demographic characteristics of screened population
Clinical benefits: sensitivity and mortality decrease	Invitation: waiting time to get test, comprehensive information, individual instructions, and ways of screening announcement.	Health worker type or sex
Harms: specificity, overdiagnosis, complication risk	Accessibility: location of test/ travel time, accessibility by public transportation	
	Convenience: possibility to combine the screening with the other health programs, waiting time for the results	

The factors grouped under the framework as procedure-related, organization-related or provider/population-related, were used to develop a semi-structured interview guide to test their importance, relevance, and clarity of the concepts. Interviews lasting 50–90 minutes were conducted until data saturation with three categories of key informants (n = 23): (1) healthcare professionals of the pilot screening centers, (2) healthcare professionals not involved in screening, and (3) target population (50–69 year-old women who participated and did not participate in screening mammography). The respondents were diverse by their age, number of years of working experience, family state, frequency of healthcare use, level of education, and income. Eight of the respondents were from rural areas while others from Minsk city. The audio records were transliterated verbatim, validated, and analysed by the content analysis with Atlas.ti software (the methodology is reported in the S2 File). All the steps of qualitative analysis were either duplicated or verified by two researchers (OM, AY).

As a result of this phase we excluded the attributes consistently reported as not important by the respondents (such as overdiagnosis, specificity, health worker sex, et al.), and those reported by the respondents as not relevant for Belarus (free treatment in case the disease is identified and accessibility of public transportation). We also added emerging themes from the interviews: queue waiting time and knowing the doctor as a “good one”. The behavioral observations showed that the respondents had difficulty in perceiving mortality decreases from screening; for this reason, screening sensitivity was selected to reflect the clinical benefit of the test. We also found that some respondents had difficulty understanding the concepts of “screening” and “sensitivity” of the tests. Thus, we developed introductory cards explaining unfamiliar definitions and teaching the respondents the process of DCEs.

**Fig 1:**
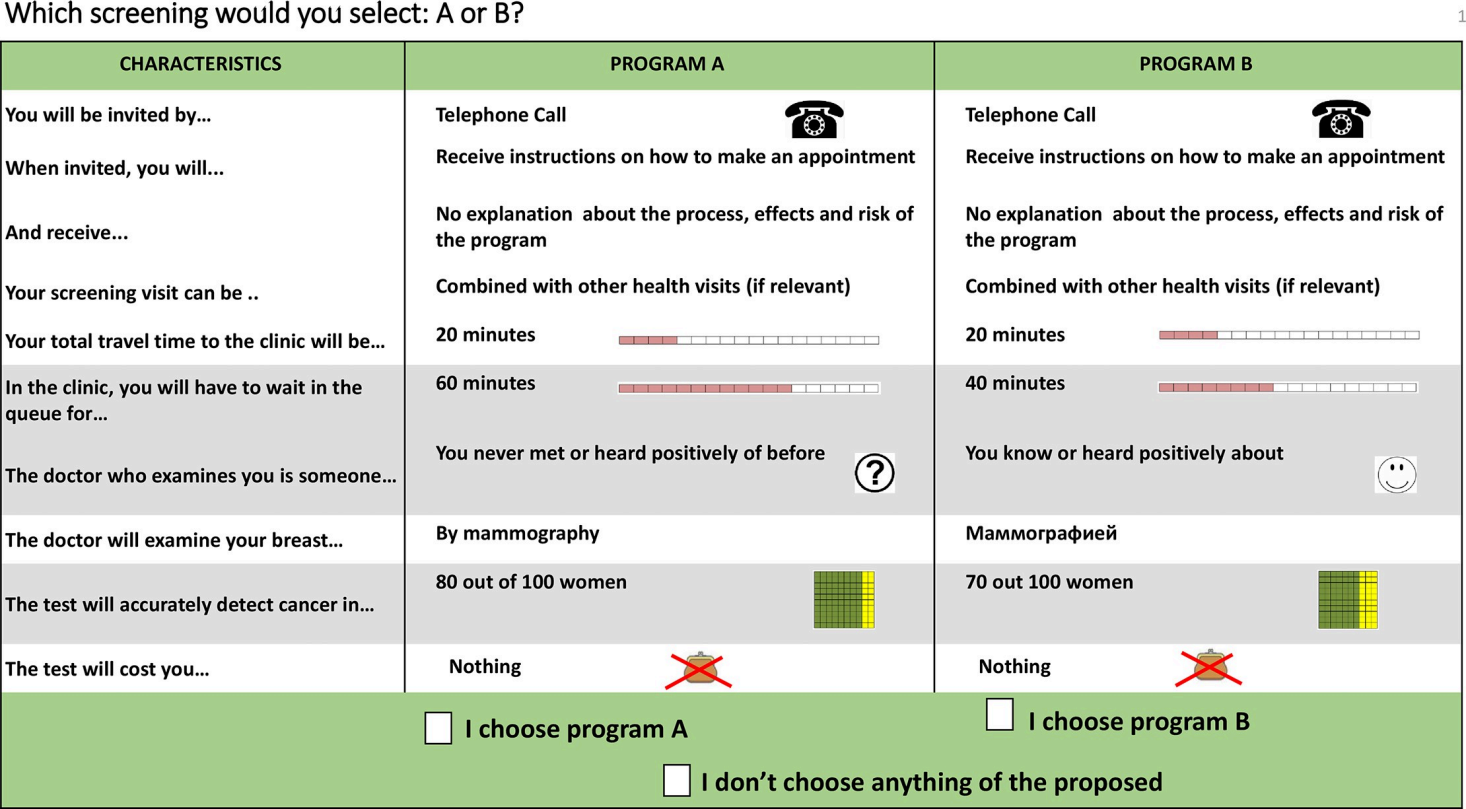
An example of the discrete choice experiment task.

We used “think aloud” (TAL) techniques to test the perception and clarity of the developed instrument, and test whether respondents considered all attributes listed when making their choice. A TAL session requires participants to verbalize their thought process during the decision making. Applying the mix of concurrent and retrospective TAL approaches, we optimized the visual design of the experiment (the font of the text and graphical location of the elements), and values of the levels for test sensitivity attribute (refer to the S2 File for the details). The final DCE design included 10 attributes described in the Table 2 with an example of the DCE task presented on Fig 1.

**Table 2:** Attributes and levels in the discrete choice experiment.

N	Attributes	Definition	Levels
1	Way of invitation	The approach a women prefers to be invited to screening	Postal letter / Telephone call
2	Possibility to arrange the appointment right away	Possibility to get the appointment arranged during the time of invitation (or fixed-appointment scheme)	Yes / No
3	Comprehensive information about screening	Receiving comprehensive information on breast cancer and screening during the invitation	Yes / No
4	Total travel time	Total travel time required for women to get from home to screening facility	20 minutes/ 40 minutes/ 60 minutes/ 90 minutes
5	Waiting time	Waiting time in healthcare facility during the screening visit	20 minutes/ 40 minutes/ 60 minutes
6	Perception of the physician as “a good doctor”	Perceiving the physician conducting the screening test as a “good” one, either because of the personal previous experience or trusted recommendation	Yes / No
7	Screening modality	Approach by which the breast cancer screening is conducted	Manual examination/ Mammography / Manually and by mammography
8	Test sensitivity	Ability of the test to detect cancer when a woman has it	60% / 70% / 80% / 90%
9	Possibility to combine the screening with other medical visits	Possibility to address several health issues within one visit to healthcare facility (for example, another screening test)	Yes / No
10	Cost of the test	Out-of-pocket costs of the screening (not reimbursed)	0 BRB / 20 BRB ^1^ / 40 BRB ^1^

^1^ 34 ID and 68 ID using purchasing power parity 2017 exchange rate (0.59).

### Experimental design

DCEs require respondents to select the preferred sets of screening program attributes. The combination of attributes and levels that respondents evaluate in a choice experiment survey is referred to as the experimental design. Our experimental design was created using a Bayesian D-efficient design optimization algorithm that was implemented in Fortran. The DCE design consisted of four versions of 18 pairwise choice sets with 10 attributes each. These four versions were simultaneously optimized, which improves the robustness and efficiency of the experimental design without resulting in a higher burden for respondents [13]. To keep the task complexity of the DCE manageable for respondents, which reduces the drop-out rate, increases choice consistency, and can avoid problems with attribute non-attendance, a design technique called attribute level overlap was used [14, 15]. Accordingly, in each choice task, seven of the 10 attributes were constrained to be presented at the same level. To increase the validity and realism of the DCE design, another design constraint was imposed to ensure that the sensitivity of CBE could be either less or equally effective but not exceed the effectiveness of mammography screening.

To reduce the required overall sample size and maximize the information obtained from the pilot studies, the results of the qualitative group analysis and subsequently those of two consecutive pilots with 40 and 150 women were used to generate the required Bayesian priors for the design optimizations. The first two DCE designs were optimized for the average population preferences but, after having obtained 190 respondents, the final DCE design was simultaneously optimized for the average population preferences as well the preferences obtained using a 2-class (latent class) logit model. Combined with the Bayesian priors, this resulted in a single DCE design that ensured statistical identification as well as optimal statistical efficiency for respondents with potentially very different preference structures. Sample size calculations as described by De Bekker-Grob et al. (2015) were used to verify identification and determine the minimally required sample size [16]. Based on these calculations, approximately 400 respondents were required to have sufficient power to obtain statistically significant results for preference parameters larger than or equal to 0.1.

### Study sample and survey administration

The study sample was stratified by share of urban and rural population and by the region of enrolment to reflect geographical representation of 50–69 year-old women in Belarus. To decrease the enrolment bias and and to ensure that the survey sample was representative by age, income, and employment status, all respondents were recruited in traumatology and burn departments of hospitals in Minsk, Brest, Vitebsk, Gomel, Grodnensk, and Mogilevsk regions. The women were enrolled if they were 50–69 years old, capable of understanding and communicating in the Russian language, provided verbal and written informed consent, and were on a recovery stage in their hospitalization. Women were excluded if they refused participation in the study or if they were considered by the interviewer incapable of formulating clear phrases and sentences verbally and in writing, had a recent history of serious breast diseases, or were hospitalized with a diagnosis that could is associated with a higher risk of breast cancer (for example, traumas related to high alcohol consumption). A written informed consent was received from each study participant prior to the interview. The process of data collection and quality control, including interviewers’ training, is presented in the S3 File.

The survey was structured as follows. First, it started with an introduction that explained the interview approach and necessary concepts, followed by a short exercise in making choices on common consumer goods (preferable fruits and telephones). To reduce the fatigue from repetitive choices, we presented the DCE in two sets of eight and ten choice tasks, with a few demographic questions included in between and with a brief health attitude survey using Likert scale statements at the end. The attitude survey included questions on beliefs in success of treatment of early detected breast cancer, fatalistic approach, and personal risk (S4 File). All the paper & pencil forms were double-entered into the database and compared for inconsistencies.

### Analysis

A latent class logit (LCL) model was used to explore respondents’ preferences for BCS. The model was chosen as the most appropriate to develop policy recommendations for different population groups rather than a single heterogeneous population. A LCL model assumes that there are c distinct sets (or classes) of respondents. Each class has its own preference parameters (i.e. β = β_1_, β_2_, .. , β_c_), which implies that preferences are assumed homogeneous within each class but allowed to be different between classes. Respondents are thus grouped based on their preferences rather than pre-specified background characteristics [17].

The optimal number of classes was determined based on a comparison of Bayesian information criterion (BIC) and consistent Akaike information criterion (CAIC) for LCL class solutions ranging from two to seven classes. After having determined the optimal number of classes, it was verified that the results were internally consistent with adequate face validity. Additionally, the average posterior probability of individual-level class-membership was calculated to obtain a quantitative measure of the quality of class-membership prediction [17].

### Policy scenarios

After having obtained the preference estimates, standard conditional logit choice share predictions were used, for each of the latent classes, to predict the BCS uptake for different policy scenarios (S5 File):

- between the existing program with CBE and screening mammography pilots;
- between population-wide screening mammography considering possible capacity constraints and CBE program;
- between hypothetical screening program in private hospitals and screening mammography pilots.

The policy scenarios were informed by the medical staff involved in screening and target population during development of the experiment (n = 23) (for the description of the sample please refer to “2.1. Selection of attributes and levels” and S2 File). CBE in Belarus is conducted at annual visits of women to the district gynecologists, and so it assumes a low waiting and travel time, familiarity with the physician, and possibility to combine the screening visits with other health reasons. The pilot mammography program is limited in geographic coverage; since current capacity is underused, the participants are actively invited both by calls and mail, and do not have long waiting time. Some women attending screening receive CBE prior to mammography. The National mammography program assumed possible capacity constrains (and so additional waiting and travel times) because of higher attendance rate of target population.

Considering that accuracy of screening is an overlapping parameter in screening programs, we excluded it from the list of attributes in policy assessments. We conducted sensitivity analyses to assess an impact of accuracy of mammography and perception of “better physicians” in private hospitals on population preferences.

## Results

### Selecting the right attributes: Qualitative results

In-depth interviews with 23 women allowed to select 10 attributes that would affect the respondents’ stated decision to attend BCS. As such, we excluded the attributes that were:

- Consistently reported as not important by the respondents to the extend being possible to affect the decision of the respondent to participate in screening (such as overdiagnosis, risk of radiation exposure, false positive results or screening test specificity, health worker sex, waiting time to get test results, test frequency, complication risk, individual versus group instructions, and type of healthcare facility);
- Considered by the respondents as not relevant for Belarus (such as an access to free treatment and accessibility by public transportation).

For instance, while the respondents wanted to be informed on screening related harms, none of these harms (within the ranges reported in systematic reviews [2]) affected their stated decision to participate in a program when the definitions of harms were explained to the respondent and correctly repeated back to the interviewer. In particular, in reasoning their choices women stated that:

- Radiation exposure with mammography screening is minimum comparatively to the other experiences (meaning Chernobyl explosion and an obligatory annual X-ray for all working population);
- False-positive test results are not important since the correct diagnosis will be revealed with the future investigation;
- Overdiagnosis would not affect the screening decision since a woman would not be able to know if she is overdiagnosed or not.

### Description of the study participants

From 490 women invited, 434 agreed to participate and 428 completed the entire survey (resulting in 89% response and 1.4% drop-out rate). The geographical distribution of the enrolled population (150 in Minsk, 64 in Brest, 55 in Vitebsk, 65 in Gomel, and 50 in Grodnensk and Mogilevsk respectively) as well as the proportion of urban versus rural population, and population with university education or above was representative in Belarus [18]. The descriptive statistics of enrolled population are presented in the Table 3. More than half of the respondents knew at least someone who had a medical history of breast cancer, with 15% of women having a relative with the history of this disease. Around two thirds of women had a BCS visit during the last year. Additionally, almost half of the women at least once in a lifetime received mammography.

**Table 3:** Characteristics of the enrolled population.

Characteristics	Categories	Number of women (%)
Healthcare users (number of visits/last 6 months)^1^	Rare (0)	79 (18%)
	Average (1–4)	260 (60%)
	Frequent (> 4)	92 (22%)
Use paid healthcare services	Within the last 6 months	120 (28%)
Have relative(s) with breast cancer	Yes	65 (15%)
Have acquaintance(s) with breast cancer	Yes	235 (55%)
Was screened within the last 12 months	Yes	302 (70%)
Have experience with mammography	Yes	200 (46%)
Practice breast self-examination (at least once during 3 months)	Yes	263 (61%)
Live alone	Yes	101 (23%)
Are employed	Full day	262 (61%)
	Partially	20 (5%)
	Doesn’t work	147 (34%)
Have university degree or above	Yes	132 (31%)
Live in the city/town	Yes	350 (81%)
Have low-income^2^	Yes	157 (36%)
Are aged, years	50–54	142 (33%)
	55–59	137 (32%)
	60–64	77 (18%)
	65–69	17%)

^1^ Not including the current hospitalization

^2^ Family income is less than 500 BYR (848 ID) per month

Regarding breast cancer perception, 64% of women believed that treatment of early-detected breast cancer has a high probability of success. At the same time, 15% and 13% of women respectively reported agreement with the statements “I will never get sick to breast cancer” or “I don’t want to know about the diagnosis if I have cancer”, while only 7% of women considered their breast cancer risk to be higher than of the other women. Almost half of women (49%) stated that they do not postpone addressing for healthcare services when they have any health issues.

### Relative attribute importance in the different latent classes

The latent class logit model with four classes was selected by the CAIC coefficient, selecting the point where adding additional latent classes no longer contributed to a meaningfully lower value (CAIC 2 Latent classes = 13,112, CAIC 3 Latent classes = 12,929, CAIC 4 Latent classes = 12,769, CAIC 5 Latent classes = 12,766). The opt-out option was selected in 19% of all scenarios among all latent classes. While second and third latent classes were quite similar in their prediction, we selected this model to reach the optimal statistical results. The results of the latent class model with three classes are presented in the S1 Table.

Class-membership prediction versus fourth class showed statistically significant difference in a proportion of frequent healthcare users in the first and third classes, proportion of women living alone in all three classes, proportion of women with previous mammography experience (lifetime experience) in the second and third classes, and proportion of women frequently practicing breast self-examination in the second class. The socio-demographic characteristics of population (education, employment state, place of habitation) could not predict the membership to the particular latent class; therefore, we report the model with no covariates.

The estimated coefficients in the model were significant in most cases. The signs of the coefficients were, conform expectations, negative for time, costs, and “doctor trust” components (meaning that women preferred the BCS programs free of charge, with low waiting and travel time, and knowing that the screening physician is a “good one”), and positive for all the other attributes (Table 4). Women in all four classes were primarily affected by the perceived reliability of the text (sensitivity and screening method) and costs (Table 4).

**Table 4:** Results of the latent class model.

Attributes	Latent Class 1					Latent Class 2					Latent Class 3					Latent Class 4				
	Utility	SE	P	95% CI		Utility	SE	P	95% CI		Utility	SE	P	95% CI		Utility	SE	P	95% CI	
Telephone invitation (vs. mailed letter)	0.131	0.080	0.104	-0.027	0.289	-0.239	0.12	0.231	-0.63	0.152	0.774	0.168	0.000	0.444	1.104	0.207	0.192	0.281	-0.169	0.583
Being able to get the appointment right away (vs. being instructed how to do it)	0.179	0.083	0.031	0.016	0.341	0.054	0.146	0.713	-0.232	0.339	-0.117	0.119	0.322	-0.350	0.115	0.033	0.172	0.848	-0.304	0.369
Detailed information on screening (vs. no information)	0.500	0.103	0.000	0.299	0.702	0.373	0.157	0.017	0.065	0.680	0.189	0.119	0.113	-0.044	0.421	-0.687	0.211	0.001	-1.101	-0.273
Possibility to combine screening with other health visits (vs. no possibility)	0.841	0.106	0.000	0.634	1.049	0.0821	0.167	0.623	-0.246	0.410	0.511	0.123	0.000	0.27	0.752	-0.0261	0.187	0.889	-0.393	0.341
Travel time 40 min (vs. 20 min)	-0.1828	0.075	0.015	-0.329	-0.036	-0.303	0.160	0.059	-0.617	0.011	-0.267	0.134	0.046	-0.529	-0.005	-0.391	0.209	0.061	-0.801	0.019
Travel time 60 min (vs. 20 min)	-0.079	0.085	0.352	-0.246	0.087	-0.998	0.181	0.000	-1.352	-0.644	-1.352	0.166	0.000	-1.677	-1.027	-0.006	0.225	0.997	-0.447	0.434
Travel time 90 min (vs. 20 min)	-0.493	0.090	0.000	-0.671	-0.316	-2.180	0.215	0.000	-2.6	-1.758	-2.018	0.232	0.000	-2.472	-1.563	-0.002	0.231	0.992	-0.456	0.451
Waiting in the queue 40 min (vs 20 min)	0.043	0.090	0.635	-0.134	0.220	-0.323	0.159	0.042	-0.634	-0.012	-0.513	0.128	0.000	-0.763	-0.263	0.145	0.212	0.494	-0.270	0.560
Waiting in the queue 60 min (vs 20 min)	-0.060	0.081	0.455	-0.219	0.098	-0.737	0.205	0.000	-1.138	-0.335	-1.391	0.178	0.000	-1.74	-1.042	0.121	0.211	0.567	-0.293	0.536
Not knowing the doctor as “good” (vs knowing)	-0.652	0.080	0.000	-0.808	-0.496	-0.128	0.145	0.377	-0.412	0.156	-0.384	0.121	0.001	-0.62	-0.148	-0.479	0.205	0.019	-0.881	-0.078
Screening by mammography (vs. manual examination)	1.458	0.12	0.000	1.224	1.693	2.104	0.251	0.000	1.611	2.596	1.402	0.239	0.000	0.934	1.87	1.626	0.443	0.000	0.759	2.494
Screening by mammography and manual examination (vs. manual examination)	1.853	0.129	0.000	1.600	2.106	2.422	0.293	0.000	1.848	2.997	1.874	0.251	0.000	1.381	2.367	1.778	0.475	0.000	0.847	2.708
Sensitivity of the test is 70% (vs 60%)	1.098	0.103	0.000	0.896	1.300	1.442	0.236	0.000	0.980	1.904	0.685	0.195	0.000	0.303	1.067	0.730	0.292	0.012	0.158	1.302
Sensitivity of the test is 80% (vs 60%)	2.131	0.120	0.000	1.895	2.367	2.435	0.282	0.000	1.881	2.988	1.418	0.208	0.000	1.012	1.825	1.451	0.400	0.000	0.667	2.236
Sensitivity of the test is 90% (vs 60%)	3.002	0.160	0.000	2.689	3.314	3.614	0.328	0.000	2.970	4.257	1.600	0.224	0.000	1.159	2.037	1.597	0.451	0.000	0.713	2.48
Cost of the test is 20 BRB (vs 0 BRB)	-0.936	0.086	0.000	-1.104	-0.768	-0.426	0.16	0.000	-0.740	-0.112	-0.907	0.142	0.000	-1.186	-0.629	-3.188	0.263	0.000	-3.703	-2.673
Cost of the test is 40 BRB (vs 0 BRB)	-1.658	0.104	0.000	-1.863	-1.454	-2.187	0.231	0.000	-2.640	-1.733	-1.158	0.162	0.000	-1.476	-0.839	-5.514	0.500	0.000	-6.494	-4.534
Opt out	-0.841	0.200	0.000	-1.233	-0.450	2.194	0.528	0.000	1.158	3.229	2.452	0.470	0.000	1.530	3.374	0.259	0.784	0.741	-1.278	1.796

Abbreviation: BRB–Belarus Rubbles; CI–confidence interval, P–probability; SE–Standard error.

The first class represented the largest share of the respondents (56%). Besides the main drivers—perceived reliability and costs—the respondents’ choices in the first class were impacted by having trust in a physician, receiving detailed information, and possibility to combine their screening visit with other visits to healthcare facility. Women in second class (16% of population) had just a few factors important besides perceived test reliability and costs, mainly related to waiting and travel time. For women in the third class (18%) perceived reliability of screening was important much less than for women in other classes, though they were also concerned by the convenience factors (travel and waiting time, being invited by telephone call, being able to combine screening with other healthcare visits, and having a trusted doctor). The fourth class included only 10% of the respondents. This group was not sensitive to any other factors besides costs, perceived reliability and screening information. While this group is in general supportive to screening, it is very price sensitive.

### Preferences to policy scenarios

The results of the assessment of population preferences to three policy scenarios are reported in the Table 5.

**Table 5:** Policy scenarios.

Levels	Current program (CBE)	Pilot MM (Minsk)	National MM screening^1^^,^^2^	Paid optimal^2^
Invitation by the post Letter		Y	Y	N
Invitation by the post telephone Call	Y	Y	N	Y
Instructions on how to make an appointment	Y	Y	Y	Y
Opportunity to arrange your appointment right away	N	N	N	Y
No explanation about the process, effects and risk of the program	Y	Y	N	N
A clear explanation about the process, effects, and risks of the program	N	N	Y	Y
Visit is related only to early detection of breast cancer	N	Y	Y	N
Visit may be combined with other health visits	Y	N	N	Y
Average travel time, min	20	20	40	20
Average waiting time, min	20	20	40	20
Unknown doctor	N	Y	Y	Y
Screening by manual examination	Y	N	N	N
Screening by mammography	N	Y	Y	Y
Screening manually and by mammography	N	Y	N	Y
Price of the screening (ID 2018)	0	0	0	68

Abbreviations: CBE–clinical breast examination; ID–International dollars; MM–mammography; N–no, Y–Yes.

^1^Population-wide screening with considered capacity restriction caused by screening expansion

^2^Hypothetical screening that could potentially be implemented within private hospitals

Women in Belarus had strong preferences for pilot mammography program comparing to existing CBE (90% on average). The lowest preference for the pilot screening mammography versus CBE was among women in the first latent class (86%), versus up to 92–99% among women in the other three classes. In a sensitivity analysis assuming 70% sensitivity of mammography versus 60% sensitivity of CBE, the preference for the latter increased to 95–100% in all four classes, while with sensitivity of 80% the preference reached 98–100%.

If the capacity constraints (such as only non-combined mammography would be available, travel and waiting time will increase, and women are invited by mail) affect the mammography screening program during its expansion to a nation-wide level, this would primarily affect the first, third and fourth latent classes where only 55%, 30% and 51% of women would prefer screening mammography under such conditions resulting to 55% of women on average preferring such screening program comparing to CBE.

A hypothetical scenario where private clinics (assuming a short waiting time, immediate appointment, and combined mammography with CBE) would be also included into the system of the national healthcare screening, would be of particular interest to women in the first and third latent class (47% and 36% of respondents would prefer this policy scenario to pilot screening mammography), though none of women in the fourth class and only 16% of the second class would prefer this option. On average, 35% of women would prefer this option to screening in public setting while 57% of women would prefer mammography screening at private clinics to CBE. In a sensitivity analysis assuming higher sensitivity of mammography in private versus public clinics (90% versus 80%), the preference to screening at private clinics increased to 38–68% in the first three latent classes. If we assume that population has higher trust in mammologists working in private clinics (the attribute “familiarity with the doctor”), the preference to private clinics will be higher in the first and third latent classes (63% and 45% respectively).

**Fig 2:**
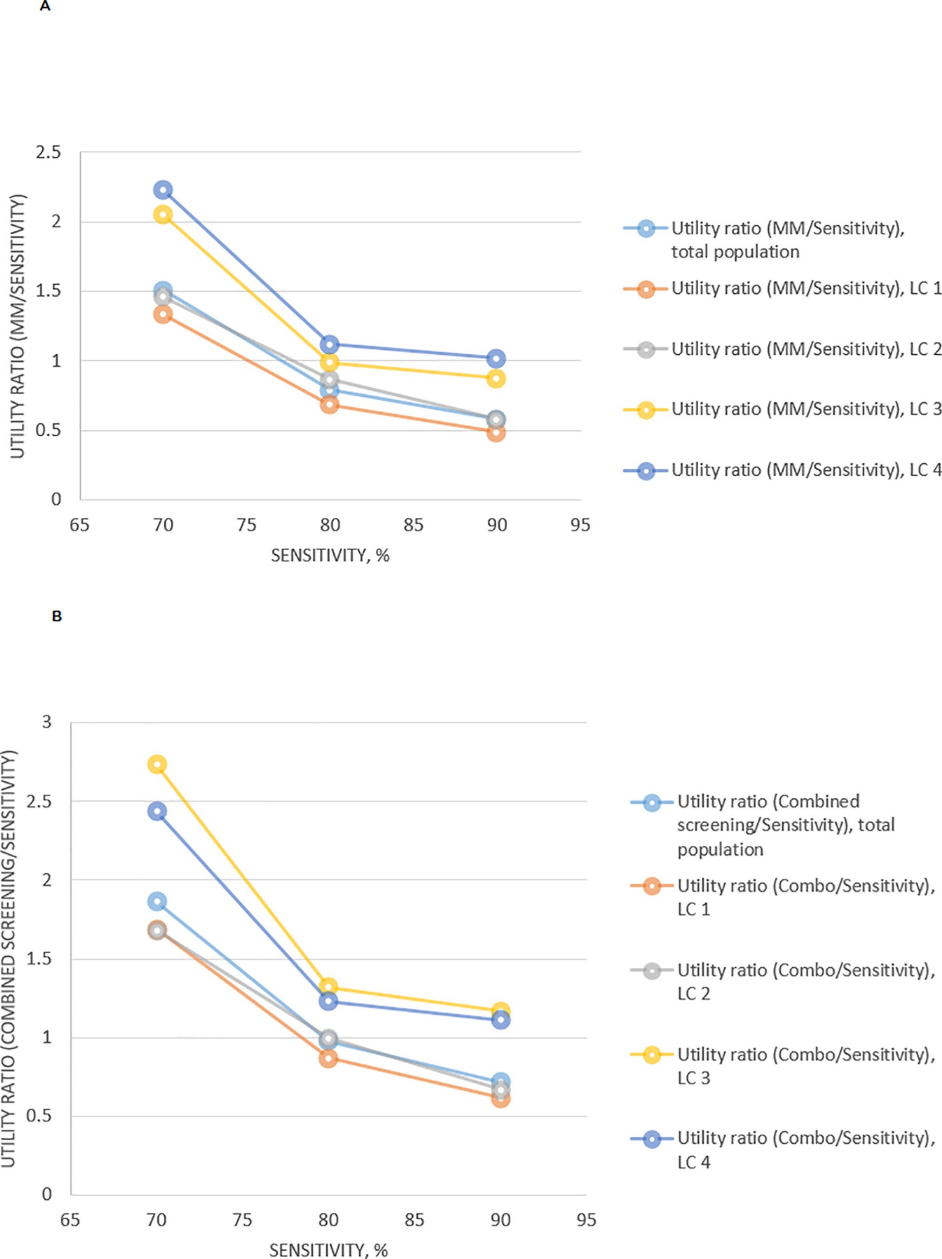
Utility of screening method and test sensitivity. (a) Screening mammography as a solo intervention, (b) Screening mammography in combination with clinical breast examination. LC–latent class, MM–mammography.

In all four classes women preferred combined screening (mammography with CBE) versus mammography as the only intervention. The importance of screening modality was higher with lower sensitivity of screening. When sensitivity was more than 80%, women on average gave more importance to sensitivity rather than screening approach, though women of third and fourth latent classes (28%) gave more importance to combined screening even with the stated sensitivity of test in 90% (Fig 2).

## Discussion

Our study showed that women in Belarus preferred mammography as a BCS program: 86–99% of the sample predicted to choose this approach instead of existing CBE. Applying a DCE to analyse women’s preferences for BCS we demonstrated that the respondents are highly sensitive to perception of the accuracy of screening. The importance of subjective characteristics of accuracy (screening modality) in stated choice of women was lower with higher values of objective factors (sensitivity of screening). The respondents’ preferences for BCS were also highly sensitive to its costs. Systematic reviews of randomized and observational studies have also confirmed an impact of removal of financial barriers on increase in BCS participation rate [19–21]. While BCS in Belarus is free of charge, the target population should be familiar with screening accessibility not to perceive it as an attendance barrier.

At the same time, among the population of 50–69 year old women around 75% was sensitive to organizational characteristics of screening, such as possibility to combine their screening with other health visits, getting detailed information on screening, being invited by a phone call, or having a trusted doctor. Besides, one-third of women are sensitive to time parameters. This can become a concern when screening will be extrapolated from pilots to a nation-wide level, and capacity constrains could result in travel requirements and waiting lines. While screening centres are easily accessible within the cities, the access to health facilities for rural inhabitants is more restricted and can decrease attendance rate among the most deprived individuals who are of the higher risk for breast cancer mortality [22]. These results are mainly consistent with the other studies using the same approach to evaluate preferences to cancer screening [3,6, 23, 24]. The latent class model with four classes was able to improve segmentation of respondents into groups with similar preferences. While cost was a significant attribute in all four classes, using the latent class analysis allowed us to identify the subgroup of women (around one tenth of population) for whom the costs and perceived reliability of screening were the only influential factors,—this population could not be distinguished using mixed logit model.

By comparing the other classes to this subgroup, we identify that women the most affected by capacity constraints (third latent class) would have a higher number of frequent healthcare users, livings alone and with experience of previous mammography. Systematic reviews demonstrated an impact of positive or negative experience with mammography screening on women’s attendance decision [19, 20, 25]. Thus, women participating in DCE could be impacted by factors affected their previous screening visit. Being married was associated with higher re-attendance rate in the review of Soler-Michel, 2005 [20]; similarly in our study the women in the fourth latent class, were in general positive about screening and had statistically different proportion of women living with the families, comparing to the other three classes. The reviews consider no or limited effect of being a frequent medical user on BCS attendance rate [19,20]; the difference in high healthcare use in the first and third versus fourth latent classes could be related to the opportunity costs considered by the respondents or to the chance findings.

Another observation of our study is that women in Belarus are in general favourable to screening: most of them stated the desire to participate if screening is highly effective and free. The population is also favourable to mammography, considering it more effective than CBE. Even more women prefer a combined program of screening mammography with CBE, which for some women (28%) is even more important than stated sensitivity of screening. This finding could be explained by an impact of personal beliefs on perceived effectiveness of screening, e.g. “the more services the better the effect”, what is in line with the findings in other studies on women’s perception related to cancer screening [26,27]. Another possible explanation could be related to feeling by the respondents the more personalised attitude with the combined screening (women, 53: “if the doctor touched my breast, it shows he cares”). While substantial evidence confirms no impact on breast cancer mortality decrease in screening with CBE combined with mammography comparing to mammography as the only intervention [28,29], through in-depth interviews we identified that some women believe in better detection of cancer with combined screening or value the combined screening as a more personalised approach. Similarly, the utility values of combined screening were higher than for screening mammography proposed as the only intervention in all latent classes. If screening mammography as the only screening intervention is provided, the target population should be well informed that the supplement screening approach would not improve screening accuracy.

Belarus has a taxation based decentralized healthcare system. The private sector expenditures of health equal to 29% of total health expenditures [30], and are mainly related to out-of-pocket payments [30] (mainly for drugs, but also related to paid services in public health centres). Besides, private market of diagnostic centres, functioning on fee-for-service bases, is highly developed in the country [30]. Our results predict that this private network could be included into the national screening program. Even though medical help in Belarus is widely accepted as free of charge, almost one third of the respondents used paid services at least once during the last six months. Our policy scenarios showed that if private hospitals are able to provide more individual-based approach (such as better explanations on screening, combining mammography with CBE, and the screening visit with other health visits), some women would prefer to attend these facilities. Considering that a large proportion of patients already choose to pay for diagnostic tests, attracted by advertisements and pleasant environment [30], the policy implication of our finding would encourage use of paid services in public and private health establishments.

While in general our findings are similar to those in other countries, we also observed some striking differences in the results. First of all, in contrast to Sicsic et al (2018)[3], our exploratory part revealed a consistent undervalue of harms, such as overdiagnosis, radiation exposure and false-positive outcomes of the screening test, to the extent that none of these characteristics was significant to be included into the experiment. The online survey of 1,000 respondents has showed that acceptability of overdiagnosis in cancer screening is variable and significantly higher for breast than bowel cancer, though lower for people aged 50 or over [21]. In our qualitative part of the study we recorded that while women were willing not know about overdiagnosis, the rate of overdiagnosis did not impact their stated preferences to attend the screening, if women understood the concept well.

Similarly, in contrast to the other DCE research on preferences to attributes of BCS in Malawi [24], neither health worker sex no type were considered to be important in our study; though there are significant differences between two countries—Belarus and Malawi—regarding the population characteristics, such as the level of education, habitation, and health beliefs.

Latent class models are rarely used in health economics, though their merits are underestimated [31]. In our study, the latent class model allowed to explore preferences to BCS attributed without complexity of models with individual heterogeneity. In a sensitivity analysis, we also estimated preferences using the mixed-mixed logit model of De Blasi et al. (2010)[32], which is a latent class logit model that accommodates preference heterogeneity within each latent class. Hardly any heterogeneity was found within each of the latent classes, which confirmed our initial choice for a latent class logit model.

While our study applied a prudent approach to develop the DCE and analyse the results, it has some limitations. Firstly, Belarus retains a commitment to the principle of universal access to health care financed through taxation [30], thus including any cost components into the survey implement potential perception bias, since some respondents may have difficulty to imagine paying for services which they confidently know are free. Meanwhile, we considered it necessary for costs to be a component of the DCE design to explore the potential of using private healthcare facilities for BCS program. Secondly, the sample enrolment was conducted in traumatology and burn departments of regional hospitals. Even though this strategy was selected specifically because it was perceived as the least biased amongst other feasible approaches, and there was no indication of bias in terms of difference among the respondents by their habitation or education level, it is obviously not possible to rule out that the composition of the sample was not entirely representative of the overall population in other aspects. Thirdly, our findings may be skewed towards more motivated sample because of the difference in preferences of responders and non-responders.

## Conclusions

Women in Belarus are heterogeneous in their preferences for screening. In general, they are willing to trade off convenience factors in BCS organization for higher quality and cheaper screening tests. Women also tend to prefer mammography as a screening method and largely disregard the screening related harms. Meanwhile, women also prefer combined BCS to single mammography, even though the clinical evidence does not support this modality. Policy makers should ensure proper communication to women regarding mammography accuracy as a stand-alone screening approach.

## Supporting information

S1 File (DOCX)

S2 File (DOCX)

S3 File Process of data collection and quality control. (DOCX)

S4 File Attitude survey on believes in breast cancer treatment and personal risk. (DOCX)

S5 File Development of policy scenarios. (DOCX)

S1 Table Results of the latent class model with three classes. (DOCX)
