# Supplementary material for: Population preferences for breast cancer screening policies: Discrete choice experiment in Belarus
Source: PLoS One. 2019 Nov 1;14(11):e0224667. doi: 10.1371/journal.pone.0224667 (PMC6824571; doi:10.1371/journal.pone.0224667)
Supplement: S1 File — (DOCX) [file pone.0224667.s001.docx]

# S1 File. Literature review

The development of the discrete choice experiment (DCE) to identify women’s preferences to breast cancer screening (BCS) in Belarus was preceded by an extensive literature review using a two-step process: (a) a review of studies applying conjoint analysis in BCS, and (b) a review of factors impacting women’s preferences in low- and middle-income countries (LMICs).

## Review of studies applying conjoint analysis

A recent systematic review on stated preferences for cancer screening by Mansfield et al (2016) [1] reported only one study [2] on preferences to BCS among general population (except studies on genetic testing). The results of the review were updated with the systematic search in Pubmed via Medline from 2013 to April 2017 using the following string line: (breast AND cancer) AND ((discrete choice experiment) OR (conjoint analysis) OR (best worst scaling) OR (preferences)) AND ((early detection) OR screening OR mammography OR (breast AND examination)). The search identified 346 abstracts, out of which two more studies using DCE in Malawi [3] and conjoint analysis in Japan [4] were included. Additionally, targeted search was conducted to retrieve studies using conjoint analysis but not identified through Pubmed search; because of the lack of relevant studies possible attributes also were extracted from three relevant studies of the other designs.

## Systematic review of studies on factors affecting breast cancer screening decisions in low- and middle-income countries

Considering expected differences in population preferences to screening depending on the socio-economic status, additional systematic search was conducted to identify any studies on women’s preferences to screening in LMICs.

The global electronic databases PubMed, Embase, and Scopus and the regional databases (Table A2) were searched in English, Russian, French, Portuguese and Spanish languages.

The search of the literature published from 2004 and above was conducted on 17-18^th^ of August 2017 with the search words listed in the Table A2. Titles and abstracts were screened and full reports of potentially relevant studies were obtained. The articles were included if they focus on: (a) BCS; (b) LMICs; (c) presents original research; (d) women older than 50 years; (e) preferences to BCS or factors that impact desire of women to participate in screening. Studies of both qualitative and quantitative designs were included. The data extracted were analyzed by content analysis using Atlas.ti software by the first reviewer and verified by the second author. Screening of 383 abstracts led to inclusion of 34 full-texts.

**Table A2. Search words used in systematic literature review in international and regional databases**

| **Search words** | **N retrieved** |
| --- | --- |
| **Pubmed** | |
| (((((((((((((((((((((((((((((((((("albania"[MeSH Terms] OR "albania"[All Fields]) OR ("algeria"[MeSH Terms] OR "algeria"[All Fields])) OR ("bahamas"[MeSH Terms] OR "bahamas"[All Fields])) OR ("bangladesh"[MeSH Terms] OR "bangladesh"[All Fields])) OR ("republic of belarus"[MeSH Terms] OR ("republic"[All Fields] AND "belarus"[All Fields]) OR "republic of belarus"[All Fields] OR "belarus"[All Fields])) OR ("belize"[MeSH Terms] OR "belize"[All Fields])) OR ("botswana"[MeSH Terms] OR "botswana"[All Fields])) OR ("brazil"[MeSH Terms] OR "brazil"[All Fields])) OR ("bulgaria"[MeSH Terms] OR "bulgaria"[All Fields])) OR ("cambodia"[MeSH Terms] OR "cambodia"[All Fields])) OR ("cameroon"[MeSH Terms] OR "cameroon"[All Fields])) OR ("colombia"[MeSH Terms] OR "colombia"[All Fields])) OR ("cuba"[MeSH Terms] OR "cuba"[All Fields])) OR ("fiji"[MeSH Terms] OR "fiji"[All Fields])) OR ("india"[MeSH Terms] OR "india"[All Fields])) OR ("iran"[MeSH Terms] OR "iran"[All Fields])) OR ("iraq"[MeSH Terms] OR "iraq"[All Fields])) OR ("jamaica"[MeSH Terms] OR "jamaica"[All Fields])) OR ("jordan"[MeSH Terms] OR "jordan"[All Fields])) OR ("kazakhstan"[MeSH Terms] OR "kazakhstan"[All Fields])) OR ("kenya"[MeSH Terms] OR "kenya"[All Fields])) OR Lao[All Fields]) OR ("malaysia"[MeSH Terms] OR "malaysia"[All Fields])) OR ("mexico"[MeSH Terms] OR "mexico"[All Fields])) OR ("morocco"[MeSH Terms] OR "morocco"[All Fields])) OR ("philippines"[MeSH Terms] OR "philippines"[All Fields])) OR ("serbia"[MeSH Terms] OR "serbia"[All Fields])) OR ("sri lanka"[MeSH Terms] OR ("sri"[All Fields] AND "lanka"[All Fields]) OR "sri lanka"[All Fields])) OR ("syria"[MeSH Terms] OR "syria"[All Fields])) OR ("syria"[MeSH Terms] OR "syria"[All Fields] OR ("syrian"[All Fields] AND "arab"[All Fields] AND "republic"[All Fields]) OR "syrian arab republic"[All Fields])) OR ("tunisia"[MeSH Terms] OR "tunisia"[All Fields])) OR ("turkey"[MeSH Terms] OR "turkey"[All Fields])) OR ("ukraine"[MeSH Terms] OR "ukraine"[All Fields])) OR ("zimbabwe"[MeSH Terms] OR "zimbabwe"[All Fields])) AND (((("mammography"[MeSH Terms] OR "mammography"[All Fields]) AND Title/Abstract[All Fields] OR (("breast neoplasms"[MeSH Terms] OR ("breast"[All Fields] AND "neoplasms"[All Fields]) OR "breast neoplasms"[All Fields] OR ("breast"[All Fields] AND "cancer"[All Fields]) OR "breast cancer"[All Fields]) AND ("diagnosis"[Subheading] OR "diagnosis"[All Fields] OR "screening"[All Fields] OR "mass screening"[MeSH Terms] OR ("mass"[All Fields] AND "screening"[All Fields]) OR "mass screening"[All Fields] OR "screening"[All Fields] OR "early detection of cancer"[MeSH Terms] OR ("early"[All Fields] AND "detection"[All Fields] AND "cancer"[All Fields]) OR "early detection of cancer"[All Fields]))) AND Title/Abstract[All Fields] OR (("breast"[MeSH Terms] OR "breast"[All Fields]) AND ("physical examination"[MeSH Terms] OR ("physical"[All Fields] AND "examination"[All Fields]) OR "physical examination"[All Fields] OR "examination"[All Fields]))) AND Title/Abstract[All Fields] OR breast screening[Title/Abstract]) AND ("2004/01/01"[PDAT] : "3000/12/31"[PDAT]) | 50 |
| (скрининг) AND рак Filters: Publication date from 2004/01/01 | 0 |
| (((postage AND cancer AND seine ) OR Mammographie)) AND (algeria OR morocco OR senegal OR tunisia) Filters: Publication date from 2004/01/01 | 0 |
| (cribado AND cáncer AND pecho) Filters: Publication date from 2004/01/01 | 0 |
| (triagem AND câncer AND peito) Filters: Publication date from 2004/01/01 | 0 |
| **Embase** | |
| (((albania OR algeria OR bahamas OR bangladesh OR belarus OR belize OR botswana OR brazil OR bulgaria OR cambodia OR cameroon OR colombia OR cuba OR fiji OR india OR iran OR iraq OR jamaica OR jordan OR kazakhstan OR kenya OR lao OR malaysia OR mexico OR morocco OR philippines OR serbia OR sri) AND lanka OR syria OR syrian) AND arab AND republic OR tunisia OR turkey OR ukraine OR zimbabwe) AND ('mammography':ab,ti OR '(breast cancer screening)':ab,ti OR '(breast examination)':ab,ti OR '(breast screening)':ab,ti) AND ([article]/lim OR [article in press]/lim OR [conference abstract]/lim OR [conference paper]/lim OR [editorial]/lim OR [erratum]/lim OR [letter]/lim OR [note]/lim OR [short survey]/lim) AND [female]/lim AND [embase]/lim AND [2004-2017]/py | 287 |
| скрининг AND рак | 0 |
| cribado AND cáncer AND pecho | 0 |
| triagem AND câncer AND peito | 0 |
| ABS ( ( ( dépistage AND cancer AND sein ) OR mammographie ) ) AND ( algeria OR morocco OR senegal OR tunisia ) | 0 |
| **Scopus** | |
| ALL ( albania OR algeria OR bahamas OR bangladesh OR belarus OR belize OR botswana OR brazil OR bulgaria OR cambodia OR cameroon OR colombia OR cuba OR fiji OR india OR iran OR iraq OR jamaica OR jordan OR kazakhstan OR kenya OR lao OR malaysia OR mexico OR morocco OR philippines OR serbia OR sri AND lanka OR syria OR syrian AND arab AND republic OR tunisia OR turkey OR ukraine OR zimbabwe ) AND ABS ( ( mammography OR ( breast AND cancer AND screening ) OR ( breast AND examination ) OR ( breast AND screening ) ) ) AND PUBYEAR > 2004 | 9 |
| скрининг AND рак | 0 |
| cribado AND cáncer AND pecho | 0 |
| triagem AND câncer AND peito | 0 |
| ABS ( ( ( dépistage AND cancer AND sein ) OR mammographie ) ) AND ( algeria OR morocco OR senegal OR tunisia ) | 2 |
| **Eastern Mediterranean: Index Medicus for the Eastern Mediterranean Region** | |
| "breast " | 18 |
| **LILAC/IBECS** | |
| cribado AND cáncer AND pecho | 0 |
| triagem AND câncer AND peito | 22 |
| triagem AND câncer AND peito | 7 |
| (tw:(triagem AND câncer AND peito)) AND (instance:"regional") AND ( limit:("female") AND year_cluster:("2012" OR "2015" OR "2010" OR "2011" OR "2013" OR "2009" OR "2006" OR "2008" OR "2014" OR "2005" OR "2004" OR "2007" OR "2016")) AND (instance:"regional") AND ( db:("LILACS" OR "IBECS")) | 29 |
| **IMSEAR** | |
| breast cancer | 10 |
| Query: Abstract:breast and Default:cancer and Default:screening   Limits: Adult; Middle Aged; Aged; Aged, 80 and over; Female; Humans | 3 |

## Results

The extensive review resulted to extraction a list of factors which were identified as significant to impact the desire of women to attend a breast cancer screening program in low- and middle income settings (Table A2).

**Table A3. Factors impacting the decision of women to attend screening by the results of systematic search for the literature**

| **Category** | **Factors of impact (reference number in the database)** |
| --- | --- |
| **Screening effect** | |
| Effect or believe/disbelieve in effect | Confidence [5], level of perceived health believe [6], accuracy (how good the test telling that you have cancer) [2, 3], benefits of MM such as "finding cancer early" [7, 8], “peace in mind from the normal result" [8]; risk of dying [9], "don't believe improve chance for survival" [10], "risk of dying" [10]. |
| Harms or believe/disbelieve in harms | Specificity ("false alarm") [8], radiation fear [6, 11-13], "MM is risky" [7] |
| **Provider characteristics** | |
| Health worker type/ qualification | No strong preferences for doctors vs nurse [3], staff attitude /satisfaction [2, 3, 9, 11], staff technical skills [3]. |
| Health worker sex | Generally female preference [3, 4, 7, 14], while some may prefer male [3] |
| **Facility /organizational characteristics** | |
| Facility of the screening | Mobile health units vs hospital (Brazil [15], Malawi [3], Japan [4]), private vs public hospitals [3], availability of equipment and medication [3], lack of capacity [3, 7], work-based screenings [4] |
| Affordability and accessibility | Screening costs [3, 7, 11, 16], access to free treatment [7], distance and capacity [3], societal or health insurance [4, 5, 12, 13, 17, 18] |
| Invitation and information | Way of invitation [9, 19, 20], requirement for comprehensive information on screening [2, 3, 14, 17, 20, 21], source of information (media [5, 7, 11, 13, 14, 20, 22], family and friends [3, 8, 20], healthcare provider [3, 5, 8, 13, 20, 23, 24]) |
| Pre-screening organization | Instructions approach (group instruction [25], size of class [3]), appointment convenience (evening time, weekend, possibility to get it) [2-4, 7, 11], appointment reminder [6, 11, 19], time till appointment [2-4] |
| Time/ convenience parameters | Possibility to combine with the other health visits [14], location/travel time [2, 3, 7, 15, 26], accessibility by public transportation [7, 19], total screening time [4, 12], time to result [2, 3], screening time [4, 27], waiting time [2-4, 7] |
| Test characteristics | Breast cancer detection strategy [3], discomfort or pain during the test [3, 6, 9, 12], test frequency [3] |
| **Individual characteristics** | |
| Socio-demographic characteristics | Age (important [4, 5, 11, 13-15, 18, 20, 22-24, 26, 28, 29] or not important [17, 19], household composition [4], place of living [9, 12, 23], religion [16], employment [4, 13, 18], education (higher with higher education [5, 10-13, 15, 16, 18, 20-22, 28-30], or indifferent [13, 28], income [11, 13, 15, 19], regular healthcare users [3, 13, 28], perceived health [4] |
| **Screening experience and perceived risk** | |
| Screening experience | Previous mammography screening (no impact [18], has impact [14]), previous clinical breast examination (CBE) had no impact [11], breast self-examination impacts CBE (15) |
| Disease history | Family history of breast cancer [4, 20, 21], history of breast diseases [13, 18, 23, 29], history of other cancers [21, 29], breast cancer in surrounding [14] |
| Risk perception and fear | High personal risk perception [3, 6, 23], low personal risk perception (believe I am healthy") [3, 4, 10], cancer fear/ anxiety [3, 6, 9, 10, 12, 23] |
| Psycho-social reasons | Health concern, lack of motivation, busy, bothersome [3-7, 9, 11, 12, 14, 18, 19, 24]; stigma [7], shame [2, 3, 7, 11, 12, 16] |
| Lack of knowledge | Negative impact [3, 5-7, 9, 11, 13, 14, 16, 22, 25, 30, 31], no difference [19, 21] |

Combining the results of the both literature reviews, the interview guide was developed which included:

1. 21 plausible factors of impact on population preferences to breast cancer screening were identified, such as: (1) efficacy (sensitivity - the probability that the test found cancer when there is cancer or the reduction in risk of cancer mortality); (2) specificity (the probability that the test will not find cancer when there is no cancer); (3) overdiagnosis; (4) discomfort or pain during the test; (5) complication risk; (6) screening costs; (7) access to free treatment in case the disease is identified; (8) type of facility where the test was preformed; (9) health worker type; (10) health worker sex; (11) screening time; (12) waiting time to get test results; (13) location of test/ travel time; (14) accessibility by public transportation; (15) test frequency (time till the next screening visit); (16) possibility to combine with the other health programs (ex. together with cervical cancer screening visit or general physical examination); (17) breast cancer detection strategy; (18) comprehensive information on screening and sources of information; (19) individual versus group instructions; (20) ways of screening announcement; (21) waiting time for the results.
2. Additional questions covering importance of the factors not included into the main guide (risk perception, fear, psych-social reasons, knowledge, motivation, etc.).
3. Demographic questionnaire covering the socio-demographic factors that could potentially impact the preferences.

## References

1. Mansfield C, Tangka FK, Ekwueme DU, Smith JL, Guy GP, Jr., Li C, et al. Stated Preference for Cancer Screening: A Systematic Review of the Literature, 1990-2013. Preventing chronic disease. 2016;13:E27. Epub 2016/02/27. doi: 10.5888/pcd13.150433.

2. Gerard K, Shanahan M, Louviere J. Using stated preference discrete choice modelling to inform health care decision-making: A pilot study of breast screening participation. Applied Economics. 2003;35(9):1073-85.

3. Kohler RE, Lee CN, Gopal S, Reeve BB, Weiner BJ, Wheeler SB. Developing a discrete choice experiment in Malawi: eliciting preferences for breast cancer early detection services. Patient preference and adherence. 2015;9:1459-72.

4. Tsunematsu M, Kawasaki H, Masuoka Y, Kakehashi M. Factors affecting breast cancer screening behavior in Japan--assessment using the health belief model and conjoint analysis. Asian Pacific journal of cancer prevention : APJCP. 2013;14(10):6041-8.

5. Dündar PE, Özmen D, Öztürk B, Haspolat G, Akyildiz F, Çoban S, et al. The knowledge and attitudes of breast self-examination and mammography in a group of women in a rural area in western Turkey. BMC Cancer. 2006;6. doi: 10.1186/1471-2407-6-43.

6. Baysal HY, Gozum S. Effects of health beliefs about mammography and breast cancer and telephone reminders on re-screening in Turkey. Asian Pacific journal of cancer prevention : APJCP. 2011;12(6):1445-50.

7. Pourfarzi F, Fouladi N, Amani F, Ahari SS, Roshani Z, Alimohammadi S. Factors Affecting Preferences of Iranian Women for Breast Cancer Screening Based on Marketing Mix Components. Asian Pacific journal of cancer prevention : APJCP. 2016;17(8):3939-43.

8. Hoffman RM, Elmore JG, Pignone MP, Gerstein BS, Levin CA, Fairfield KM. Knowledge and values for cancer screening decisions: Results from a national survey. Patient education and counseling. 2016;99(4):624-30.

9. Dundar PE, Ozyurt BC, Erdurak K. Sociodemographic determinants of nonattendance in a population-based mammography screening program in the city of Manisa, Turkey. The Scientific World Journal. 2012; doi: 10.1100/2012/816903.

10. Gyrd-Hansen D, Sogaard J. Analysing public preferences for cancer screening programmes. Health economics. 2001;10(7):617-34.

11. Yildirim AD, Özaydin AN. Sources of breast cancer knowledge of women living in Moda/Istanbul and their attendance to breast cancer screening. Meme Sagligi Dergisi / Journal of Breast Health. 2014;10(1):47-56. doi: 10.5152/tjbh.2014.1762.

12. Ersin F, Polat P. Examination of factors affecting women’s barrier perception to participate in breast cancer screenings in a region affiliated with a family health center in Turkey. Turkish Journal of Medical Sciences. 2016;46(5):1393-400. doi: 10.3906/sag-1502-89.

13. Ozmen T, Soran A, Ozmen V. Comparison of barriers against mammography screening in socioeconomically two contrarious populations. Journal of Clinical Oncology. 2016;34.

14. Tuzcu A, Bahar Z, Gözüm S. Effects of interventions based on health behavior models on breast cancer screening behaviors of migrant women in Turkey. Cancer Nursing. 2016;39(2):E40-E50. doi: 10.1097/NCC.0000000000000268.

15. Vieira RAC, Lourenço TS, Mauad EC, Moreira Filho VG, Peres SV, Silva TB, et al. Barriers related to non-adherence in a mammography breast-screening program during the implementation period in the interior of São Paulo State, Brazil. Journal of Epidemiology and Global Health. 2015;5(3):211-9. doi: 10.1016/j.jegh.2014.09.007.

16. Islam RM, Bell RJ, Billah B, Hossain MB, Davis SR. Awareness of breast cancer and barriers to breast screening uptake in Bangladesh: A population based survey. Maturitas. 2016;84:68-74. Epub 2015/12/01. doi: 10.1016/j.maturitas.2015.11.002.

17. Aksoy YE, Turfan EC, Sert E, Mermer G. Barriers on Breast Cancer Early Detection Methods. The journal of breast health. 2015;11(1):26-30. Epub 2015/01/01. doi: 10.5152/tjbh.2014.2296.

18. Maral I, Irem Budakoǧlu I, Özdemir A, Bumin MA. Factors affecting participation in population-based mammography screening. Trakya Universitesi Tip Fakultesi Dergisi. 2010;27(2):122-6. doi: 10.5174/tutfd.2008.01139.1.

19. Khaliq W, Visvanathan K, Landis R, Wright SM. Breast cancer screening preferences among hospitalized women. Journal of women's health (2002). 2013;22(7):637-42. doi: 10.1089/jwh.2012.4083.

20. Aker S, Oz H, Tuncel EK. Practice of Breast Cancer Early Diagnosis Methods among Women Living in Samsun, and Factors Associated with This Practice. The journal of breast health. 2015;11(3):115-22. Epub 2015/01/01. doi: 10.5152/tjbh.2015.2547.

21. Peacock S, Apicella C, Andrews L, Tucker K, Bankier A, Daly MB, et al. A discrete choice experiment of preferences for genetic counselling among Jewish women seeking cancer genetics services. British journal of cancer. 2006;95(10):1448-53. doi: 10.1038/sj.bjc.6603451.

22. Dişcigil G, Şensoy N, Tekin N, Söylemez A. Breast health: Knowledge, behaviour and performance in a group of women living in the Aegean region. Marmara Medical Journal. 2007;20(1):29-36.

23. Guvenc I, Guvenc G, Tastan S, Akyuz A. Identifying women's knowledge about risk factors of breast cancer and reasons for having mammography. Asian Pacific journal of cancer prevention : APJCP. 2012;13(8):4191-7.

24. Oyur Çelik G, Malak AT, Öztürk Z, Yilmaz D. Analysis on the condition of women in post menopausal period on their application of breast self-examination and having mammography and pap smear screening. Anatolian Journal of Clinical Investigation. 2009;3(3):159-63.

25. Seven M, Akyüz A, Robertson LB. Development of a service model for a breast cancer screening program and evaluation of its effectiveness. Asia-Pacific Journal of Clinical Oncology. 2014;10:21. doi: 10.1111/ajco.12332.

26. Hayran M, Abali H, Kilickap S, Mentes T, Aksoy H, Kemik A, et al. Socio-demographic parameters in screening for breast cancer: Lessons from a population-'based women's Health Project held in a province in Turkey. Journal of BUON : official journal of the Balkan Union of Oncology. 2010;15(4):726-31.

27. Griffith GL, Edwards RT, Williams JM, Gray J, Morrison V, Wilkinson C, et al. Patient preferences and National Health Service costs: a cost-consequences analysis of cancer genetic services. Familial cancer. 2009;8(4):265-75. Epub 2008/09/30. doi: 10.1007/s10689-008-9217-5.

28. Cabioglu N, Ozmen V, Ozaydin AN, Gulluoglu BM, Unalan PC, Gorpe S, et al. Survey on a pilot mammographic screening programme in Istanbul, Turkey. European Journal of Cancer, Supplement. 2010;8(3):243. doi: 10.1016/S1359-6349(10)70656-X.

29. Perera JC, Peiris V, Wickramasinghe DP, De Zoysa I. Predictors in breast cancer screening behaviors of South Asian women. Asia-Pacific Journal of Clinical Oncology. 2016;12(2):e229-e33. doi: 10.1111/ajco.12168.

30. El Mhamdi S, Bouanene I, Mhirsi A, Sriha A, Ben Salem K, Soltani MS. Women's knowledge, attitudes and practice about breast cancer screening in the region of Monastir (Tunisia). Australian Journal of Primary Health. 2013;19(1):68-73. doi: 10.1071/PY11123.

31. Türk R EK, Terzioğlu F, Taşkın L. . An Example from the Rural Areas of Turkey: Women Breast Cancer Risk Levels and Application and Knowledge Regarding Early Diagnosis- Scan of Breast Cancer. Eur J Breast Health. 2017;13:67-73.
