# Supplementary material for: Population preferences for breast cancer screening policies: Discrete choice experiment in Belarus
Source: PLoS One. 2019 Nov 1;14(11):e0224667. doi: 10.1371/journal.pone.0224667 (PMC6824571; doi:10.1371/journal.pone.0224667)
Supplement: S2 File — (DOCX) [file pone.0224667.s002.docx]

# S2 File. Supporting information 2

## In-depth interviews with key informants: development of experimental design

To develop the experimental design we conducted 13 in-depth interviews with leads of the screening units, gynaecologists, nurses and family doctors in the pilot region (Minsk and Minsk district) and 10 more with a qualitative sample of 50-69 year old who were previously exposed to mammography and those women who have never previously participated in screening. The inclusion criteria for target population were the following: 50-69 years old, capable of understanding and communicating in the Russian language, and provided verbal and written informed consent. We applied purposeful sampling strategy aiming to identify well-informed participants among health care workers and diverse (both screening compliant and screening resistant) participants among lay population. The contacts with the requested types of participants were provide by local health administrations.

Among the included respondents (age range 49-69 years), eight were from rural areas and the others were recruited in Minsk. The respondents were diverse by their age, number of years of working experience (for healthcare professionals), family state, frequency of healthcare use, highest level of education, and income for general population. The description of participants from lay population is presented in the Table B1.

Table B1. Description of the participants in in-depth interviews, the general population (n =10)

| Characteristics | Categories | Number of women |
| --- | --- | --- |
| Healthcare users (number of visits/last 6 months) | Rare (0) |  |
|  | Average (1 - 4) |  |
|  | Frequent (> 4) | 2 |
| Use paid healthcare services | Within the last 6 months |  |
| Have relative(s) with breast cancer | Yes | 2 |
| Have acquaintance(s) with breast cancer | Yes | 8 |
| Was screened within the last 12 months | Yes | 6 |
| Have experience with mammography/ experienced mammography the last 12 months | Yes | 6/4 |
| Practice breast self-examination (at least once during 3 months) | Yes | 4 |
| Live alone | Yes | 3 |
| Are employed | Full day | 8 |
|  | Partially |  |
|  | Doesn’t work |  |
| Have high education or above | Yes | 2 |
| Live in the city/town | Yes | 7 |
| Have low-income | Yes | 2 |
| Are aged, years | 50-54 | 4 |
|  | 55-59 | 3 |
|  | 60-64 | 1 |
|  | 65-69 | 2 |

Before inclusion, the respondents confirmed correspondence to the inclusion criteria. Each participant was familiarized with the research method, its purpose, and objectives, and signed the informed consent form prior the interview. All the respondents filled in the demographic questionnaire in the end of the interview.

The inclusion criteria were the following:

1. Women 50-69 years old capable to understand and communicate in Russian language (estimated as more than 95% of population).
2. Women who have provided verbal and written informed consent and are willing and able to follow the protocol.

Participants will be excluded if at least one of the following conditions are present:

1. Women who refused to complete the study or who were considered by the interviewer incapable of formulating clear phrases and sentences verbally and in writing. .
2. Women who refused to sign the informed consent form.
3. Women who have history of breast cancer, currently have breast cancer, or serious breast diseases.

Two different semi-structured interview guides (pre-tested on 2 respondents in each group) were developed to interview the healthcare professionals and general population. The healthcare professionals were asked to provide their personal opinion on breast cancer screening, working experience with screening program, sources of knowledge, facilitators and barriers in screening attendance, characteristics of adherent and non-adherent populations, clarity of the concepts and definitions for parameters describing screening and screening outcomes, importance of each of the parameters in the final decision of women to attend. The general population was asked on personal experience related to the same explored categories; they were also presented with the developed visual materials and asked to describe the meaning of the illustrations and definitions to ensure the clarity of the provided concepts, and the importance of the provided information in their personal decision to attend the screening. The choice of semi-structured interview guide versus a structured one was argued by the flexibility of the approach, which allows elaboration of themes important to participants but may not thought by the researchers (1).

The individual interviews lasted from 50 to 90 minutes. The number of interviews was conducted until data saturation. All of the interviews were audio recorded, transcribed verbatim and double-verified for the accuracy of transcription. In addition, the notes regarding the psycho-social behavior of the respondent during the interview (hesitation, time needed to understand the concepts, etc.) were taken to fulfill the audio transcripts. The content analysis the interview transcripts was conducted using Atlas.ti software. Categories for data analysis were selected in accordance with the developed interview guide. In addition, we grouped similar appearing information into subcategories, selected in accordance with the participants’ replies under similar categories (Table B2).

As a result of this phase we excluded the attributes consistently reported as not important by the respondents to the extend being possible to affect the decision of the respondent to participate in screening (such as overdiagnosis, specificity, health worker sex, waiting time to get test results, test frequency, complication risk, individual versus group instructions type of facility where the test was preformed), those considered by the respondents as not relevant for Belarus (access to free treatment in case the disease is identified, accessibility by public transportation), and added the attributes considered by the respondents as important, but not identified as such during the previous stage (queue waiting time, knowing the doctor as a “good one”). As a result of behavioral observation, we found that the respondents had difficulty in perceiving mortality decrease from screening, thus screening sensitivity was selected to reflect the clinical benefit of the test. We also found the difficulty of the respondents to understand the concepts of “screening” and “sensitivity” of the tests. Thus, the introductory cards were developed to explain the unfamiliar definitions (such as “screening” and “sensitivity” of the test) and the interview process. The demographic questionnaire was amended to address the difficulty in perception of the questions by study participants, including the questions related to income, place of living, level of education, and family state.

**Table B2. Categories and sub-categories identified during the in-depth interviews with key informants**

| **Category** | **Screening MD**  **(n = 4)** | **MD group**  **(n = 9)** | **Population group**  **(n =10)** |
| --- | --- | --- | --- |
| **Current situation with pilot screening** | | | |
| **Information during invitation** | No information on breast cancer or screening during the invitation (5 respondents), not clear how many respondents receive written invitation (1 respondent) | | |
| **Results** | Very quick (6 respondents) | | |
| **Distance** | Close walking distance (20-45 minutes) (5 respondents) | | |
| **Waiting** | Sometimes there may be queues and difficulty to get appointment with waiting time more than a month (1 respondent) | Waiting time for more than 60 min is possible and burdensome, can affect the decision (3 respondents) | Waiting time for more than 60 min is possible and burdensome, can affect the decision (4 respondents) |
| **Process** | Clinical breast examination be done by any clinical specialist, typically done by gynecologists, lack of training for MD, some MD don’t participate in screening themselves | Don't know if there is mammography screening in assigned policlinics, do screening at work because obliged to do it (3 respondents) | Invited by GPs or during the visit to policlinics (3 respondents) |
| **Costs** | Everyone knows that treatment is free (1 respondent) versus “maybe not everyone” (1 respondent), important to clarify that screening is free (1 respondent); low capacity in policlinics, some go to the paid services (2 respondents) | Everyone knows that treatment is free. Two suggested there might be someone not 100% confident; and for screening it is better to clarify (8 respondents). | Two respondents considered treatment costs a lot (but it would not change their decision to go for screening), eight were sure it is free. All assumed screening is free. |
| **Capacity** | Gynecologists are overloaded, and other specializations are not motivated, private clinics are not informed on screening and early detection (2 respondents) | Doctors are not aware of screening themselves (2 respondents) | Doctors are not motivated (2 respondents) |
| **Importance of factors** | | | |
| **Benefit** | Sensitivity should be 98-99%, otherwise patients will be afraid to be in the remaining group (1 respondent). | Believe in effectiveness of mammography (6 respondents), "bad result is obvious immediately"(1 respondent). Think that effectiveness of screening is the most important (14 respondents), even small decrease in mortality is important (13 respondents) and may depend on the doctor (1 respondent), | |
| **Harms** | Not important for patients (3 respondents), maybe for some patients but not the majority (1 respondent) | Radiation risk, false positive and overdiagnosis are not important (16 respondents); don’t believe in overdiagnosis after the explanation (3 respondents) | |
| **Provider characteristics** | Gender and specialization are rarely an issue, may be important for some patients (don't want to go to the nurse) (2 respondents). | Prefer mammologist, but trust that educated nurse can do a good clinical breast examination (4 for, 3 against); trust to the doctor is more important, prefer the older experienced doctors (2 respondents); not influential to the extent to change the decision, but will prefer a female doctor (4 respondents) | |
| **Trust in the healthcare specialist** | Believe in “good doctor" (1 respondent), trust in doctors skills is very important (1 respondent) | Use healthcare resources because trust the doctor (4 respondents) | |
| **Facility /organizational characteristics** | | | |
| **Private hospitals** | Many patients go to private hospitals because they trust the facilities (1 respondent) | Prefer to pay for better quality (2 respondents) | |
| **Distance** | Distance to facility is important for some women (1 respondent) | Mobile units decrease waste of time (2 respondents), | Distance to facility is important for some women (1 respondent) |
| **Specialized versus local centers** | Some patients think more specialized hospitals have better doctors and equipment(1 respondent) | Trust in better equipment, less painful (2 respondents) | |
| **Appointment** | Women want to have short waiting time for an appointment (forget or change the plans), low capacity (2 respondents) | Should be in the evening for working population (2 respondents), avoiding queues is important (2 respondents) | Fixed appointment ("didn't go because there were queues and I needed to go and make an appointment"; "I don't mind to come if the date and time will be arranged" "need to go in the morning for a ticket, no time") (3 respondents), avoiding queues is important (4 respondents) |
| **Time aspects** | Short travel time this will motivate rare healthcare users(1 respondent), only slightly important since the max time to reach policlinics is 40 min walking (1 respondent); transport is easily available (1 respondent), queues may happen sometimes (1 respondent) | Important (9 respondents) or not (6 respondents), maximum tolerant travel time is 1 hour (3 respondents); transport is easily available (4 respondents); need to take a day-off from work, what may be problematic (1 respondent), time in queue is critical (8 respondents) [none of the respondents was a resident of villages outside of Minsk district] | |
| **Screening characteristics** | Maybe for some patients, but not generally (1 respondent); depends on healthcare regular use and trust to the doctor (1 respondent); One respondent believe she is able to self-identify cancers (breast examination) and so do not attend the screening | Mainly not important (5 respondents). Prefer instrumental methods (2 respondents) or combined with clinical breast examination (2 respondents), believe they are able to self-identify cancers (2 respondents) | Moderately important (3 respondents). Prefer methods combined with clinical breast examination (2 respondents), don't think that instrumental methods can mistake (2 respondents), don't see a need in mammography if they already do clinical breast examination (1 respondent) |
| **Screening frequency** | Not important for the most, especially if there is a trust in a doctor; but some will not go if too frequently because of the pain (2 respondents) | Relatively important (1 respondent), not important (6 respondents); | Not important in general, but may affect those who fear radiation (10 respondents) |
| **Description of adherent population** | High healthcare users, those who do clinical breast examination (2 respondents) | Those who do clinical breast examination, have healthy lifestyle and take care about their health (6 respondents); work-requested physical examinations (8 respondents) | Those who have healthy lifestyle and take care about their health (all in adherent group were doing regular breast examination) (9 respondents); Medical checkup is requested at work (2 respondents) |
| **Invitation** | Some don't reply to written invitations, multiple calls are more important, home telephones are rarely used (1 respondent) | Not important if it is an individual way (5 respondents); Preferred to be invitation from their doctor (1 respondent), prefer telephone (3 respondents), rarely use stationary phone (1 respondent) | Individual way of invitation is important, but no difference which call or letter (3 respondents), or prefer a call (5 respondents). Few people preferred email. Three people mentioned not using telephones or preferring ones. |
| **Information** | “The more the better" (1 respondent), use media (primarily TV) (2 respondents), “word-of-a-mouth” (1 respondent), internet (1 respondent), a lot of sources available (1 respondent), healthcare professionals always instruct on screening (1 respondent), insufficient knowledge among population (1 respondent) | Only brief is needed (1 respondent), very detailed is needed (9 respondents); brochures are desirable (2 respondents), prefer information from the doctor (1 respondent), use media (for 3 respondents, against 4 respondents), consider a lot of information is available (7 respondents), consult family member with medical education (3 respondents), consult with nurse/physician (1 respondent), doctors instruct on breast self-examination (2 respondents) | |
| **Psycho-social (perceived) characteristics** | | | |
| **Fears and concerns** | Don’t want to know if she herself has a disease so does not go for cancer screening (1 respondent), lack of thinking about their own health (1 respondent) | Concern about own health (4 respondents), previous breast diseases (3 respondents), perceived the personal risk as lower than average or what to think that way (4 respondents), fear of cancer (6 respondents), don’t want to think of the health problems if any (3 respondents), busy/ not concerned (4 respondents). | |
| **Beauty** | Breast saving message is important: "women like for everything to be beautiful", "breast is their treasure" (2 respondents) | Breast saving message is not important: "now breast elimination is not so scary, - a lot of opportunities for a prosthesis" (1 respondent) | Breast saving message is important: "Even if in time, still the breast will be cut" (1 respondent) |
| **Believes** |  | Breast cancer screening should start from 30 or 40 (3 respondents), don’t think cancer can ever happen to her (1 respondent), breast cancer is easy to cure (6 respondents), breast cancer is caused by hormone misbalance (2 respondents) or trauma (4 respondents). | |

The Legend: Screening MD – a group of medical specialists involved directly in screening; MD – a group of medical specialists, not involved directly in screening; Population group – group of women either with or without screening experience.

## Attributes and levels in the discrete choice experiment

The DCE design included 10 attributes:

- **Way of invitation:** an important factor for the majority of the respondents and by the results of literature review; the parameter is diversely implemented in different pilots of mammography screening in Belarus.
- **Possibility to arrange the appointment right away**: the parameter was given additional value as a self-emerged (not included into the semi-structured interview guide) topic from the interviews with several respondents (see the category “Appointment” in the Table B1).
- **Comprehensive information about screening:** an important factor for the majority of the respondents and by the results of literature review; the parameter is diversely implemented in different pilots of mammography screening in Belarus.
- **Total travel time:** an important parameter for eight respondents and in the results of the literature review; the parameter is affected by healthcare capacity.
- **Waiting time:** an important parameter for eight respondents and in the results of the literature review; the parameter is affected by healthcare capacity.
- **Perception of the physician as “a good doctor”:** the parameter was given additional value as a self-emerged (not included into the semi-structured interview guide) topic from the interviews with several respondents (see the category “Trust in the healthcare specialist” in the Table B1).
- **Screening modality:** an important factor for the respondents and by the results of literature review; the parameter is diversely implemented in different pilots of mammography screening in Belarus with some physicians performing clinical breast examination before directing to mammography screening while others directing the women without clinical breast examination.
- **Test sensitivity:** an important parameter for the majority of the respondents and in the results of the literature reviews.
- **Possibility to combine the screening with other medical visits:** an important parameter for some of the respondents and in the results of the literature reviews.
- **Cost of the test: an important parameter by the results of the literature review. Was included to address use of paid services in state or private facilities** (see the category “Private hospitals” in the Table B1).

## In-depth interviews with key informants: testing the experimental design

We used think aloud’’ (TAL) technique to test the perception and clarity of the developed instrument, and whether the respondents consider all attributes listed when making the choice.

The pilot study included ten 50-69 years old women in total. In a TAL approach the participants were asked to verbalize their thought process during decision making. There are two types of think aloud: concurrent and retrospective. It is recommended that a combination of the two should be used wherever possible. Concurrent think aloud is where people are asked to verbalise what they are thinking as they complete a certain task. Retrospective think aloud asks people to describe what they were thinking after the task has been completed. We applied a mixed approach: the respondents were asked to think-aloud for two or three choices, and then to reflect back after this. If the respondent was silent for a period of time, she was reminded to keep thinking aloud. Experiences of the TAL process were then discussed with participants who also filled in a demographic questionnaire to test the validity and perception of the instrument.

The interviewer recorded the interviews and take detailed notes on the participant’s responses and whether or not she had difficulty with particular choice sets, wording, or images. In particular, the interpreted the lengthy pauses made by the respondents and their visual signs (e.g. brining the presented slide closer to the face) as poor readability of the draft instrument. The final experimental design, including the text formulation and images presentation, was constructed considering the results of piloting the instrument.

As a result of this step, we changed the visual design of the experiment, by moving the optout option from left corner of the card to the right, increasing the font of the text on each card and editing some wording. We decreased the levels for the attribute “sensitivity of the screening” from 70-80-90-95 to 60-70-80-90 since some of the women considered the accuracy of more than 90% to be difficult to achieve and so where choosing irrationally. We also introduced a bond so that accuracy of clinical breast examination could not be higher than of mammography screening to make the scenarios believable for the respondents.

## Quality assurance

We predicted the validity, reliability and quality of the demographic questionnaire using SQP survey quality predictor system (<http://sqp.upf.edu>). Since the data on Belarus were not available, we used Ukraine as a reference country to Belarus to evaluate the quality of each question.

The in-depth interviews were conducted by the experienced interviewer, trained in Qualitative Research Methods and with the previous experience in leading the focus groups and interviewing healthcare professionals and general population. We applied the following methods to control the quality of the qualitative part of the project:

1. Triangulation. We compared the results received through the interviews with healthcare professionals, interviewed women, and categories extracted from the literature. We also discussed an effect of personal characteristics such as age, education, economic level, and professional status on the data. We reviewed possible intellectual, personal and interviewers’ biases.
2. Data saturation and attention to negative cases. We conducted interviews in one-by-one basis extracting the relevant information. If any contradictory data are retrieved, the interviewer discussed these elements with the respondent to explore the reasons for inconsistency. If the contradictions remain unexplored after the interview, the interview guide was relevantly updated. All the changes inserted into the guide were recorded.
3. Perspective incorporation. To ensure that opinion of all relevant subgroups of the respondents are considered, we had a purposeful sample including population of (1) low income; (2) working and retired; (3) single and married; (4) age groups of: younger than 55, 55-65, older than 65; (5) low-educated and high-educated groups.

1. Gill P, Stewart K, Treasure E, Chadwick B. Methods of data collection in qualitative research: interviews and focus groups. Bdj. 2008;204:291.
