# Supplementary material for: Population preferences for breast cancer screening policies: Discrete choice experiment in Belarus
Source: PLoS One. 2019 Nov 1;14(11):e0224667. doi: 10.1371/journal.pone.0224667 (PMC6824571; doi:10.1371/journal.pone.0224667)
Supplement: S3 File — (DOCX) [file pone.0224667.s003.docx]

# S3 File. Process of data collection and quality control

## Recruitment of the sample

According to the protocol, the target study sample (aimed n = 400) was stratified by urban/rural areas and the region of enrolment to reflect geographical representation of 50-69 year-old women in Belarus. To decrease the enrolment bias, all the respondents were contacted in the emergency departments of the regional hospitals of six administrative regions (Minsk, Brest, Vitebsk, Gomel, Grodnensk, and Mogilevsk) (Table C1).

**Table C1. Planned enrolment of Belarus women in different regions of the country (n = 400)**

| Region of Belarus | Urban area,  N of women | Rural area,  N of women | Total,  N of women |
| --- | --- | --- | --- |
| Minsk region | 117 | 26 | 143 |
| Brest region | 41 | 17 | 58 |
| Vitebsk region | 39 | 11 | 50 |
| Gomel region | 46 | 14 | 60 |
| Grodnensk region | 33 | 11 | 44 |
| Mogilevsk region | 36 | 9 | 45 |

The sampling strategy was selected by consensus among the research group and two stakeholders involved in pilots of mammography screening programs of the Ministry of Health Belarus. The other sampling approaches (such as work environments, markets/shops, parks or residential areas) were considered with the higher risk of sampling bias related to the differences in employment and/or income, low response rate, or poor quality of the interview processes.

The inclusion criteria were the following:

1. Women 50-69 years old capable to understand and communicate in Russian language.
2. Women who have provided verbal and written informed consent and are willing and able to follow the protocol.

Participants will be excluded if at least one of the following conditions are present:

1. Women considered by the investigator to be unwilling or unable to complete the study or unable to comprehend or complete the study questionnaires.
2. Women who are unwilling to comply with the protocol or who are unable to complete the questionnaires.
3. Women who have history of breast cancer, currently have breast cancer, or serious breast diseases.
4. Women who were hospitalized with the diagnosis that might be associated with higher risk of breast cancer.

To each of the respondents, the interviewers explained the purpose of the study and gathered both verbal and written informed consent from each study participant prior to any study procedures being performed. Consent was documented with the woman’s dated signature on a Consent Form along with the dated signature of the interviewer conducting the consent discussion. A copy of the signed and dated consent form was given to the woman before she could participate in the study.

All of the respondents replied on the 18 choice designs. The structure of the experiment split into the blocks was the following:

(1) Introductory slides and explanation of the experiment;

(2) First eight choice sets of the experiment;

(3) “Distraction” slides and demographic questionnaire;

(4) Ten choice sets of the experiment;

(5) Questions on the experience participating in the experiment.

No insurance for population is provided since the study has observational design. The cost associated with a time lost is estimated on a level of near 10EUR. These costs were not be reimbursed directly. As a gratitude a shopping card for this value was provided to participants. The value of the gift card provided was considered as very small to impact participants’ replies or affect their desire to participate in the study despite the discomfort or lack of interest, what was acknowledged by the interviewed respondents.

## Data analysis and record keeping

All the quantified data were typed in the Electronic Data Collection system (EDC) created in Excel. Data were typed into EDC by the interviewers from paper informational sources either collected on sites or retrieved through content analysis. The second interviewer duplicated all data entry. In addition, each of the interviewer maintained a subject enrolment log. This log recorded all approached potential participants, reasons for inclusion or exclusion, and refusal rate. All the documented records were transferred by the interviewers to the researchers. The hard copies of the study documents are stored by the site center in department of cancer control NN Alexandrov National Cancer Centre of Belarus, Minsk, Republic of Belarus. The electronic version of the documents were provided to the members of the research team. All protocol-required information that is originally recorded elsewhere (e.g., demographic data), was transcribed into the EDC as directed by the study procedures.

## Study Documentation and Records Retention

Study documentation includes informed consent forms with signature pages, data collection forms, data handling and entry guidelines, source documents, correspondence and regulatory documents (e.g., signed protocol and amendments, Independent Ethics correspondences and approvals).

Source documents include all recordings of observations including all audio files recording the interviews as well as notes from the interviews, demographic questionnaires and any other reports and records necessary for the evaluation and reconstruction of the study. Whenever possible, the original recording of observation should be retained as the source document. They will be retained for 5 years after the completion of the study at the site in Belarus. Data generated by this study are considered confidential by the investigator, except to the extent that it is included in a publication.

## Training

The Principal Investigator (PI) had an extensive experience conducting qualitative data collection including leading the focus groups and in-depth interviews and passed the online training on Qualitative Research Method. The PI (O. Mandrik) and the second investigator (A. Yaumenenka), who conducted the qualitative data collection, passed the online course “Protecting Human research Participants” by the National Institute of Health (USA).

All the interviewers were the employees of the NN Alexandrov National Cancer Centre of Belarus, Minsk, which is one of the several centers placing mammography screening in Belarus. While all of them are experienced with the screening methods and approaches, they were required to pass the trainings on Research Ethics and Protection of Research Participants (based on materials of the course “Protecting Human Research Participants” by the National Institute of Health translated into Russian) and the study protocol before participating in the study (the training log is kept on site). Besides passing the test to confirm the theoretical background, each interviewer had minimum five accompanied interviews until one of the key researchers (OM or AY) considered the skills of the interviewer sufficient to start the data collection process.
