# Supplementary material for: Population preferences for breast cancer screening policies: Discrete choice experiment in Belarus
Source: PLoS One. 2019 Nov 1;14(11):e0224667. doi: 10.1371/journal.pone.0224667 (PMC6824571; doi:10.1371/journal.pone.0224667)
Supplement: S4 File — (DOCX) [file pone.0224667.s004.docx]

**S4. File. Attitude survey on believes in breast cancer treatment and personal risk**

On a scale from 1 («Strongly disagree») до 5 («Strongly agree»), indicate the correctness of these statements.

|  | Strongly disagree | |  | Strongly agree | |
| --- | --- | --- | --- | --- | --- |
| Breast cancer in most cases is successfully treated on early stages | 1 | 2 | 3 | 4 | 5 |
| I think that I will not get sick to breast cancer | 1 | 2 | 3 | 4 | 5 |
| I don’t want to know if I have breast cancer | 1 | 2 | 3 | 4 | 5 |
| Even if detected early, in most cases it is useless to treat breast cancer | 1 | 2 | 3 | 4 | 5 |
| I think that my risk to get breast cancer is higher than of the other women | 1 | 2 | 3 | 4 | 5 |
| As soon as I have health issues, I address for a medical help immediately | 1 | 2 | 3 | 4 | 5 |
