# Supplementary material for: Population preferences for breast cancer screening policies: Discrete choice experiment in Belarus
Source: PLoS One. 2019 Nov 1;14(11):e0224667. doi: 10.1371/journal.pone.0224667 (PMC6824571; doi:10.1371/journal.pone.0224667)
Supplement: S5 File — (DOCX) [file pone.0224667.s005.docx]

# S5 File. Development of policy scenarios

We assessed preferences of the population to three policy scenarios, comparing mammography pilots and hypothetical nation-wide screening program to the current clinical breast examination program and screening through private hospitals. The policy scenarios were informed by medical staff involved in screening and population (n = 23).

**Pilot mammography program**

The current pilot mammography screening program invites women to screening by both telephone call and post, though the invitation materials do not include information on breast cancer and BCS. There is an expected waiting time (3-4 months) will the appointment though since the program currently covers only specified districts and the capacity is underused, the average travel and waiting time is limited to 20 minutes. Women in pilot screening frequently go through clinical breast examination before having mammography since they attend the gynaecologist prior to screening.

**Clinical breast examination program**

The Ministry of Health of Belarus recommends women to attend gynaecologists each year while require to do so all employed females. These district gynaecologists also conduct clinical breast examination during each visit of women. Since patients are flexible with the appointments, their visit to gynaecologists may be combined with other health reasons. They do not get a written invitation to attend screening but may be reminded by phone. The breast cancer screening is conducted in local health units (“polyclinics”) what results to low travel and waiting time for women. Many women have established longitudinal relationships with their district gynaecologists, who follows up all the residents in the assigned district.

**National mammography program**

Considering that the current pilot mammography screening program is low-scaled and underused because of low familiarity of women with its availability, we assumed that escalation of the program will result to capacity constrains and so longer waiting time. We also assumed a longer travel time because of expansion of the program to more distant districts (e.g. rural areas). We assumed unfeasibility of telephone call invitation approaches on the national scale though we considered that the Ministry of Health will develop informational materials to invite women to screening. Considering that the Ministry of Health targets to substitute clinical breast examination with screening mammography, we assumed that combined screening would not be available in the future.

**Paid optimal program**

The scenario of paid optimal program included a possibility of screening in private hospitals with higher custom-focus (different invitation options, information delivery, and possibilities for combined or rapid appointments), and better perceived quality indicators (familiarity with the doctor and combined screening).
