# Supplementary material for: Population preferences for breast cancer screening policies: Discrete choice experiment in Belarus
Source: PLoS One. 2019 Nov 1;14(11):e0224667. doi: 10.1371/journal.pone.0224667 (PMC6824571; doi:10.1371/journal.pone.0224667)
Supplement: S1 Table — (DOCX) [file pone.0224667.s006.docx]

**S6 Table. Results of the latent class model with three classes**

| Attributes | Class 1 | Class 2 | Class 3 |
| --- | --- | --- | --- |
| Telephone invitation (vs. mailed letter) | 0.114 | 0.266 | 0.202 |
| Being able to get the appointment right away (vs. being instructed how to do it) | 0.175 | -0.076 | -0.126 |
| Detailed information on screening (vs. no information) | 0.405 | 0.351 | -0.392 |
| Possibility to combine screening with other health visits (vs. no possibility) | 0.699 | 0.463 | 0.095 |
| Travel time 40 min (vs. 20 min) | -0.179 | -0.250 | -0.276 |
| Travel time 60 min (vs. 20 min) | -0.056 | -1.270 | -0.288 |
| Travel time 90 min (vs. 20 min) | -0.490 | -2.205 | -0.424 |
| Waiting in the queue 40 min (vs 20 min) | 0.091 | -0.348 | -0.328 |
| Waiting in the queue 60 min (vs 20 min) | -0.036 | -0.991 | -0.510 |
| Not knowing the doctor as “good” (vs knowing) | -0.609 | -0.149 | -0.406 |
| Screening by mammography (vs. manual examination) | 1.405 | 1.694 | 1.171 |
| Screening by mammography and manual examination (vs. manual examination) | 1.786 | 1.948 | 1.617 |
| Sensitivity of the test is 70% (vs 60%) | 1.045 | 1.019 | 0.598 |
| Sensitivity of the test is 80% (vs 60%) | 2.069 | 1.818 | 1.201 |
| Sensitivity of the test is 90% (vs 60%) | 2.885 | 2.572 | 1.331 |
| Cost of the test is 20 BRB (vs 0 BRB) | -0.915 | -0.264 | -2.429 |
| Cost of the test is 40 BRB (vs 0 BRB) | -1.681 | -1.234 | -3.725 |
| Opt out | -0.616 | 2.388 | 0.936 |
| Class share | 0.604 | 0.228 | 0.168 |

The Legend: BRB – Belarus rubbles
